# Supervised Lowess normalization of comparative genome hybridization data – application to lactococcal strain comparisons

**DOI:** 10.1186/1471-2105-9-93

**Published:** 2008-02-11

**Authors:** Sacha AFT van Hijum, Richard JS Baerends, Aldert L Zomer, Harma A Karsens, Victoria Martin-Requena, Oswaldo Trelles, Jan Kok, Oscar P Kuipers

**Affiliations:** 1 Molecular Genetics, Groningen Biomolecular Sciences and Biotechnology Institute, University of Groningen, Kerklaan 30, 9751 NN Haren, the Netherlands; 2 Computer Architecture department, University of Malaga, 29071, Malaga, Spain; 3 Current address: Interfacultary Centre of Functional Genomics, Ernst-Moritz-Arndt-Universität, Greifswald 17489, Germany; 4 Current address: Molecular Cell Biology, Groningen Biomolecular Sciences and Biotechnology Institute, University of Groningen, Kerklaan 30, 9751 NN Haren, the Netherlands; 5 Current address: Department of Microbiology and Alimentary Pharmabiotic Centre, University College Cork, Western Road, Cork, Ireland

## Abstract

**Background:**

Array-based comparative genome hybridization (aCGH) is commonly used to determine the genomic content of bacterial strains. Since prokaryotes in general have less conserved genome sequences than eukaryotes, sequence divergences between the genes in the genomes used for an aCGH experiment obstruct determination of genome variations (e.g. deletions). Current normalization methods do not take into consideration sequence divergence between target and microarray features and therefore cannot distinguish a difference in signal due to systematic errors in the data or due to sequence divergence.

**Results:**

We present supervised Lowess, or S-Lowess, an application of the subset Lowess normalization method. By using a predicted subset of array features with minimal sequence divergence between the analyzed strains for the normalization procedure we remove systematic errors from dual-dye aCGH data in two steps: (1) determination of a subset of conserved genes (i.e. likely conserved genes, LCG); and (2) using the LCG for subset Lowess normalization. Subset Lowess determines the correction factors for systematic errors in the subset of array features and normalizes all array features using these correction factors. The performance of S-Lowess was assessed on aCGH experiments in which differentially labeled genomic DNA fragments of *Lactococcus lactis *IL1403 and *L. lactis *MG1363 strains were hybridized to IL1403 DNA microarrays. Since both genomes are sequenced and gene deletions identified, the success rate of different aCGH normalization methods in detecting these deletions in the MG1363 genome were determined. S-Lowess detects 97% of the deletions, whereas other aCGH normalization methods detect up to only 60% of the deletions.

**Conclusion:**

S-Lowess is implemented in a user-friendly web-tool accessible from . We demonstrate that it outperforms existing normalization methods and maximizes detection of genomic variation (e.g. deletions) from microbial aCGH data.

## Background

Array-based comparative genome hybridization (aCGH) is applied frequently to study the genomic content of closely related micro-organisms [1], microbial taxonomy and species determination [2], and the presence of microbial pathogenicity factors [1,3-5]. The differences in aCGH signals depend on systematic errors (e.g. dye effects), copy number variation, and sequence divergence between the analyzed samples [6]. Since prokaryotes generally show lower genomic conservation than eukaryotes, normalization of bacterial aCGH data is difficult.

Over the years, the following normalization methods have been described for aCGH data: total signal normalization [5,7], Lowess [8-10], normalization using background signals [11], spatial normalization [12], and mixed models of Lowess and spatial normalization [13]. Furthermore, tools have been developed for detecting gene duplications/deletions [6] and for the graphic exploration of aCGH data [10,14,15]. None of the above-mentioned methods take into account sequence divergence and thus only partly can correct for the systematic errors that occur in aCGH data. Consequently, these methods result in lower discovery of genome variations, like gene deletions or duplications (see below).

To solve this problem we first select a subset of array features corresponding to genes with minimal sequence divergence between the strains used for an aCGH hybridization. We term this subset of features likely conserved genes (LCG). LCGs can be determined with certainty when both bacterial strains used for the aCGH experiment have been sequenced, or approximated by inference if one of the strains has not been sequenced, but sequences of closely related strains (termed here reporter genomes) are available. Also, from ortholog databases one can select the most conserved gene sequences and use these as an LCG set. With subset Lowess, correction factors for systematic effects occurring in aCGH data are derived for LCGs. These correction factors are subsequently used to normalize all array features. On basis of these considerations, we developed supervised Lowess (S-Lowess) which has been implemented in user-friendly web-based software. In a controlled aCGH experiment, labeled genomic DNA of *Lactococcus lactis *IL1403 and *L. lactis *MG1363, of which the genome sequences are known, was hybridized to *L. lactis *IL1403 DNA microarray slides. We show that S-Lowess clearly outperforms existing implementations of aCGH normalization methods in detecting gene deletions with a low number of false-positives (i.e. genes falsely called being absent).

## Results

### Supervised Lowess

For normalization of bacterial aCGH data, we evaluated the application of subset Lowess [16] using a subset of conserved genes (*i.e*. likely conserved genes; LCG) which are considered to be homologous in the strains-of-interest. We propose that the sequence divergence of LCGs between the samples analyzed is minimal; enabling a more robust estimation of systematic errors in the aCGH data using the LCG array features. Below we show that after application of S-Lowess on microbial aCGH data, genome variations (e.g. gene deletions of duplications) can more reliably be determined.

**Figure 1:**
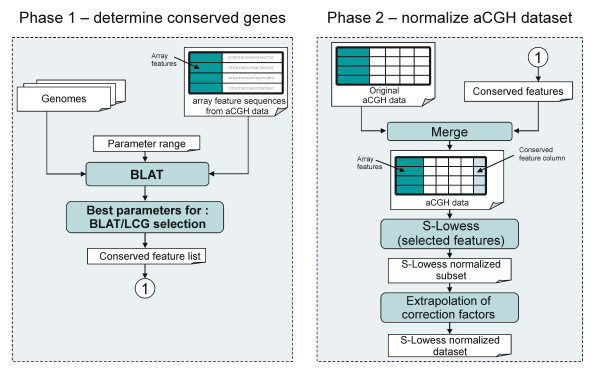
**Flow diagram of the S-Lowess procedure**. The S-Lowess procedure consists of two phases: 1) determine or upload likely conserved genes (LCG) and 2) Normalize a microarray dataset with the LCGs. In case that for phase 1 *de novo *prediction of LCGs is selected, the user has to upload microarray feature sequences and select (multiple) genomes (in this study 3 *Streptococcus *genomes). The optimal parameters for selection of LCGs from a sequence comparison using BLAT of array features versus multiple reporter genomes are difficult to predict. Therefore, selection of a LCG set is facilitated by cycling through a maximum of 2 parameters. These parameters are (a combination of two): (i) alignment length cutoff, (ii) E-value cutoff, (iii) percentage nucleotide identity cutoff, (iv) maximum number of hits within the same genome (to account for paralogous genes or duplicated genome fragments), (v) minimum number of hits across genomes (to account for gene conservation in multiple genome sequences). Those array feature sequences meeting the criteria (here in at least 2 out of three genomes a significant BLAT hit; one hit over at least 100 bp with at least 80% nucleotide identity) are marked as LCG and added to the conserved array feature list. In phase 2, the LCGs are used to normalize an uploaded aCGH microarray dataset. The result of phase 2 is a normalized dataset and diagnostic plots.

The S-Lowess procedure is outlined in Figure 1. First, an LCG set is selected from sequence comparisons between those of the microarray features and the genes in the genomes of related species. The features which demonstrate a high sequence identity with the genomes of related organisms are considered to be conserved and are selected. In the second step, the aCGH dataset is normalized by applying Lowess to the LCG features, *i.e.* S-Lowess. During this normalization, at first correction factors for the LCG features are determined by robust curve fitting. Then, the signals of all array features are normalized using these correction factors.

### The LCG set

An important step in the S-Lowess procedure is selection of array features for the LCG set. LCG were selected based on various parameters: *i.e.* sequence identity, length of identical sequence, etc. A detailed description is available in the methods section. In general, when less stringent sequence identity cut-offs are applied during LCG selection, more robust Lowess curve fits are obtained at the expense of estimation of systematic errors in the aCGH data. Subsequently, this results in less accurate detection of genomic deletions or duplications. In addition, the systematic errors in DNA microarray data are better determined by performing curve fits on grids (areas spotted by one spot pen) rather than on the whole slide (this study). As a rule of thumb, we have observed (by visual inspection of the curve fits and minimizing the coefficients of variation that curve fits based on at least 50 spots (whole slide) or 20 spots (grid-based) lead to satisfactory results.

The genes selected in the different LCG sets (see above) are shown in Figure S1 [17]. In general, for both the percentage nucleotide identity cutoff and the E-value cutoff, more stringent parameters lead to lower numbers of LCGs selected. Genes selected on the basis of BLAT E-value cutoffs were quite different from those selected by the percentage of nucleotide identity. As shown below, normalization using the LCG sets by applying these different cutoffs leads to different end results.

### Different normalization methods lead to different data distributions

**Figure 2:**
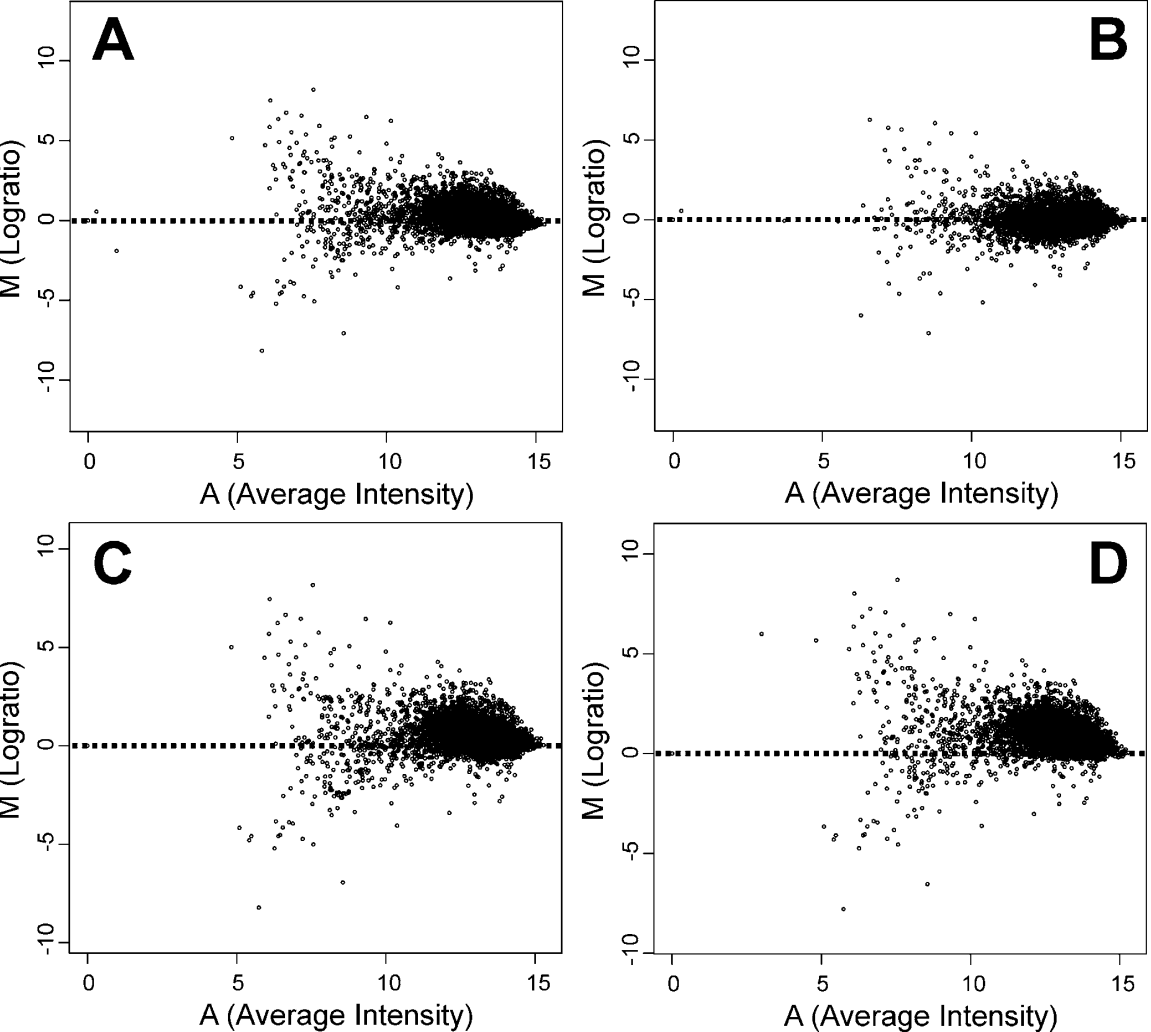
**MA-plots of aCGH data after applying different normalization methods**. The log transformed ratios of slide 11 [17] are plotted against the log transformed sum of the green (negative M values; MG1363 signals) and red (positive M values; IL1403 signals) channels. A: non-normalized data. B: grid-based Lowess normalization. C: S-Lowess normalization based on the LCG set obtained from the comparison of *L. lactis* IL1403 amplicon sequences to the ORFs of three *S. pneumoniae *strains. D: S-Lowess normalization with a stringent LCG set (99% identity over 100 bp).

In order to evaluate various normalization methodologies, we generated aCGH data in experiments in which we compared the genomic content of two sequenced lactococcal strains. A visual inspection of the comparison of S-Lowess normalized data with the data obtained from other normalization methods reveals clear differences in the distribution of spot intensities (Fig. 2). Ideally, for the aCGH comparison of *L. lactis *IL1403 and *L. lactis *MG1363, one would expect that the bulk of ratios reside above the X-axis (corresponding to a ratio of 1; signal of MG1363 over that of IL1403), since labeled *L. lactis *IL1403 DNA will, for most array features, hybridize more efficiently than those of *L. lactis *MG1363.

Grid-based Lowess normalization of the aCGH data results in an even distribution of data points along the M = 0 (a log2 transformed ratio of 0; or a normal ratio of 1) axis (Fig. 2B), which is not according to the expectation described above. After S-Lowess normalization of the aCGH data with the limited LCG set (99% nucleotide identity over 100 bp; Fig. 2D) or the realistic test case (Fig. 2C), the bulk of ratios is above the M = 0 axis, as expected.

### S-Lowess outperforms other methods in predicting genomic deletions

The performance of different normalization procedures on aCGH data in predicting genome variations was determined by comparing these to the known deletions in the genome sequence of *L. lactis *MG1363 compared to that of *L. lactis *IL1403.

**Figure 3:**
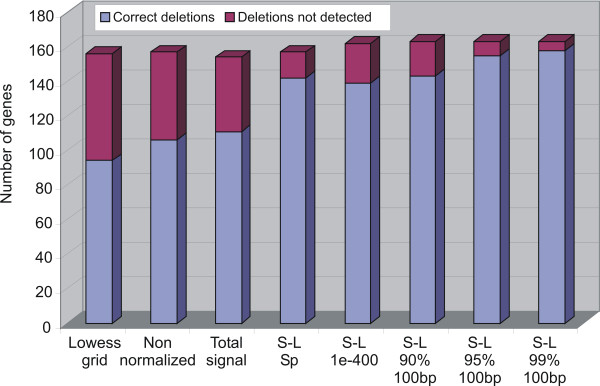
**Performance of the different normalization methods in the identification of deletions in *L. lactis *MG1363**. Blue: number of deletions correctly called (here a cutoff of 1.5 fold is used). Purple: number of deletions missed. S-L: S-Lowess. S-L Sp: S-Lowess normalization based on the LCG set obtained from the comparison of *L. lactis *IL1403 amplicon sequences to the ORFs of three *S. pneumoniae *strains. The total heights of the bars indicate the total number of amplicons for missing ORFs in *L. lactis *MG1363 with at least 5 aCGH measurements. They thus indicate the total number of missing ORFs that could be detected based on the aCGH data.

In general, the S-Lowess normalization yielded a higher number of correctly detected deletions in the *L. lactis *MG1363 genome (see Fig. 3 and [17]) compared to Lowess or total signal normalization, and non-normalized data. Application of the GENCOM method [18] to the *L. lactis *MG1363 and *L. lactis *IL1403 aCGH dataset results in a poor performance: only 45 of the over 2122 genes are divergent and the differences between both strains reported by GENCOM are approximately evenly distributed [17].

The number of false-positively scored genes being present, ranged from 37% by S-Lowess (based on a LCG set with an E-value cutoff 1 × E-100) to 3% (based on the LCG set with 99% nucleotide identity across all alignment length cut-offs) and for the non S-Lowess methods from 73% (Lowess grid-based normalization) to 45% (total signal normalization). Clearly, the use of S-Lowess with LCG sets determined on the basis of BLAT E-values gives poorer performance than using the percentage identity cut-off as basis for LCG selection (Fig. 3 and [17]). Normalization of aCGH data with the LCG set selected on the basis of comparison of *L. lactis *IL1403 array feature sequences to the ORFs of three *Streptococcus *strains gave the same number of correctly identified deletions as the LCG set based on 90% sequence identity over 100 bp with less missing genes scored as being present. It is slightly outperformed by the best case, using the LCG set based on a 99% nucleotide identity cutoff.

In this study, we focus on the genes that are absent in the genome of *L. lactis *MG1363. The lactococcal genomes have limited sequence identity (on average 85%). Therefore, many *L. lactis *MG1363 ORFs have a low sequence identity with *L. lactis *IL1403 counterparts. Due to the limited sequence identity of these MG1363 ORFs, they should be recognized as being absent in the aCGH experiment (see also Fig. S3 [17]). Furthermore, only for the S-Lowess normalized data and not for the non-normalized or Lowess grid-based normalized data a distinct pattern of clusters of large differences across the genome of *L. lactis *MG1363 is apparent (Fig. S4 [17]).

### Nucleotide identity and fold ratio are highly correlated in S-Lowess normalized data

**Figure 4:**
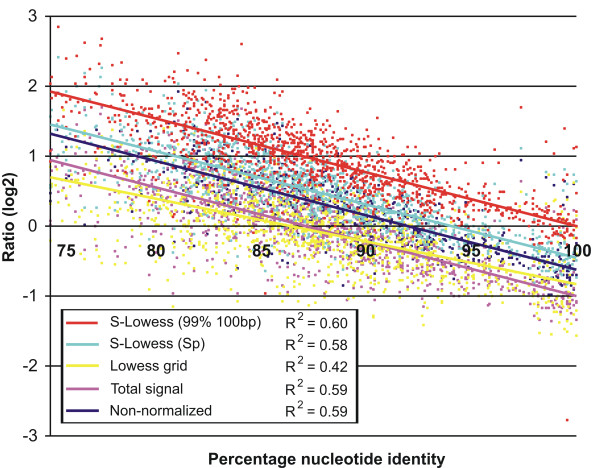
**Correlation plots of the normalized ratios (signals for labeled gDNA of *L. lactis *IL1403 over those of *L. lactis *MG1363) and the percentage sequence identity of orthologous genes in *L. lactis *MG1363 and *L. lactis *IL1403**. Sp: S-Lowess normalization based on the LCG set obtained from the comparison of *L. lactis *IL1403 amplicon sequences to the ORFs of three *S. pneumoniae *strains. The R^2 ^values indicate the quality of the regression curve fit (where higher is better).

The comparison of two bacterial strains with known genome sequences allowed determining the concordance of the ratio obtained in an aCGH experiment and the actual sequence identity between the genes in these strains. At a 100% sequence identity of *L. lactis *IL1403 array features compared to the *L. lactis *MG1363 gene sequences identical signals should be obtained, resulting in log2 ratios of 0. Normalization of of the aCGH data using the LCG features selected should therefore yield a better correspondence of fold change versus nucleotide identity. Figure 4 shows the fold ratio of a number of different normalization methods as a function of sequence identity. Based on the lower R^2 ^value the Lowess normalized data is noisiest (Fig. 4). The S-Lowess normalized aCGH data using a stringent LCG set (99% identity over 100 bp) performed as expected since the intersection with the x-axis of the regression line through the data at a log2 transformed ratio of 0 (normal ratio 1) is at 100% identity. Lower performance of the other methods is visualized by the intersection of their regression lines at lower percentages identity, in particular by total signal normalization and Lowess normalization (Fig. 4).

## Discussion

S-Lowess outperforms existing normalization methods in correctly predicting the absence of genes in dual-dye aCGH data. This result is achieved by a more accurate estimation of the systematic errors occurring in aCGH data by using genes that are likely to be conserved. Existing methods do not take differences in sequence identity into account resulting in "over fitting", which is clear from data points centered on the M-axis (e.g. for Lowess normalized data; Fig. 2B). For S-Lowess, the application of multiple genomes of related strains could increase the number of LCGs, which in turn results in an even better estimation of the systematic errors. Bacterial strain typing and detection of duplications and deletions can thus be performed more accurately by using S-Lowess.

Dong and coworkers [19] observed that, based on 83 genes, a negative trend exists between percentage sequence identity and fold-ratio between *Klebsiella pneumoniae *and *Escherichia coli *K12. They fitted two regression lines through the non-normalized data: (i) for genes with 55 – 75% nucleotide identity and (ii) for genes with 75 – 100% nucleotide identity. We also observed that lower sequence identity results in higher deviant ratios and more noise (results not shown) and, therefore, decided to only focus on curve fits for 75 – 100% sequence identity. Possibly, hybridization of DNA with lower sequence identity is less predictable and depends strongly on the length and composition of the homologous DNA sequence. The curve fits of Dong and coworkers intersect at M = 0 and 88% sequence identity, which lies well between the values we observed for total signal normalized (87%) and non normalized (92%) data (Fig. 4). One would expect the curve fit to intersect at M = 0 and 100% nucleotide sequence identity. This is only the case for S-Lowess normalized data with a stringent LCG set (i.e. 99% sequence idenity over 100-bp; see Fig. 4).

A difference in signal between samples in an aCGH experiment is due to systematic errors in the data, a difference in gene sequence identity, or the absence/duplication of a gene. In a non-isogenic transcriptome experiment, mRNA profiles of different strains are obtained by DNA microarrays. In this case, a difference in signals between samples could additionally be due to a difference in gene expression level. The S-Lowess concept could also be used to normalize such non-isogenic transcriptome data as a difference in signal for LCGs is very likely to be due to differential expression and not to difference in sequence identity. The Lowess assumption, namely that for a significant number of genes the expression is not altered, will probably hold for the LCG set, provided that the different organisms were grown and treated similarly. Performing subset Lowess normalization with a stringent LCG set should therefore produce a robust estimation of the systematic errors. For aCGH data a large fraction of the LCGs should have hybridization ratios close to 1. For non-isogenic transcriptome experiments, a fraction of the LCGs could be differentially expressed and, therefore, the curve fits should be performed with lower fractions of genes (up to 50%). This means that slide-based S-Lowess will probably be preferred for non-isogenic transcriptome normalization.

The performance of S-Lowess in a realistic worst-case scenario was estimated from normalizing aCGH data of *L. lactis *IL1403 and *L. lactis *MG1363 with a LCG set determined from taxonomically relatively close bacterial species. In this realistic case, S-Lowess clearly performed better than convential normalization methods. We also tested the performance of S-Lowess on a *Shigella *aCGH dataset [20] (Fig. S5 [17]). The genome sequence of this strain is as of yet unknown, which makes a comparison of the aCGH data to actual genome sequence data not possible. Therefore, the properties of the *Shigella *dataset after treatment with different normalization methods were compared. Clearly, the data properties for the *Shigella *dataset benefit strongly from the application of S-Lowess as we also demonstrated for the *L. lactis *aCGH dataset (Fig. 2).

The power of S-Lowess is that it can be applied to any two-dye aCGH dataset. Since aCGH experiments are performed with unknown strains, one cannot make strong assumptions as to the genetic content of these strains, making S-Lowess the method of choice to normalize data from any prokaryote aCGH experiment. The number of genome sequences is growing rapidly [21] and a further acceleration is anticipated with the use of new techniques [22]. Therefore, even better performance of S-Lowess is expected when higher numbers of more similar reporter genomes are used for the selection of conserved genes.

## Conclusion

Supervised Lowess is a new application of the subset Lowess normalization technique to microbial array-based comparative genome hybridization (aCGH) data. In particular but not exclusively, when the strains hybridized are relatively distant, no strong *a priori *assumptions can be made as to the data distribution of microbial aCGH data. A difference in signal might be due to systematic errors in the microarray data (e.g. dye effects) but could also be due to a difference in sequence between the two samples hybridized. Presently used aCGH normalization methods do not take into account this source of variation: sequence divergence between the genes of the strains hybridized. S-Lowess is unique with the property that it uses the signals of likely to be conserved genes (LCG; with minimal sequence divergence) to estimate the systematic errors in aCGH data. We demonstrate on an aCGH dataset of two distant *L. lactis *strains that S-Lowess performs superior compared to existing normalization techniques used for microbial aCGH data.

## Methods

### The S-Lowess web-tool

The supervised Lowess normalization method is new in that it uses the features of likely conserved genes (LCGs) to perform the subset Lowess normalization (see below for a detailed description). A flow diagram of the S-Lowess web-tool is presented in Figure 1. The procedure consists of two steps: (i) generate or upload an LCG set; and (ii) normalize one or more DNA microarray datasets using this LCG set.

The LCG set consists of genes that are likely to be conserved between the two samples hybridized to a microarray. Since in most cases the genome sequence of at least one of the strains hybridized to the microarray is not known, the genes that are likely to be conserved in this/these strain(s) are determined by using reporter genome sequences. These reporter genome sequences should be as close as possible to the strains hybridized. To determine the LCG set, the sequences of the array features are compared to the DNA sequence of ORFs of the selected reporter genomes by BLAT (BLAST Like Alignment Tool) [23].

When an LCG set has been selected, normalization is performed on tabulated data from a single dual-dye DNA microarray slide. The user selects from the uploaded dataset the data columns (containing the Cy3 and Cy5 signals from each feature) and the parameters to be used for normalization. The default parameters should suffice for performing a slide-based normalization of most cases. After normalization, diagnostic plots and the normalized data are presented. In addition, the percentage of LCG features (spots) with ratios outside the threshold ratio (2 by default; fewer outlying spots are better) is indicated.

### The subset Lowess algorithm

Supervised Lowess normalizes an aCGH microarray dataset using the LCG array features for the subset Lowess normalization. Subset Lowess normalization and its application for e.g. normalization of spiked-in dual dye DNA microarray data has been described before [16]. It computes the initial LogRatios (*i.e.Ri *(i = 1 ..*N*)) per slide, followed by Lowess normalization of a selected subset of conserved genes, generating a set of corrected ratios *Rc i *(i = 1*..n*, *n*<*N*) and correction factors for the subset of conserved genes used: αi = *Rc i *- *Ri*. Subsequently, the Lowess correction factors belonging to the subset of conserved genes (αi) are extrapolated to determine the correction factors βj (j = *n*+1*..N*) for the remaining genes. The correction factors are then used to adjust the log-ratios of the remaining genes. The subset Lowess normalization procedure used in this study has been implemented in the Windows software PreP [24] and a linux command line utility sl (both are available upon request).

### Bacterial strains, media and isolation of chromosomal DNA

*L. lactis *subsp *lactis *IL1403 (Lac-; Prt- ; plasmid-free derivative of IL594) [25] and *L. lactis *subsp *cremoris *MG1363 (Lac-; Prt- ; plasmid-free derivative of NCDO712) [26] were grown statically at 30°C in M17 broth [27] supplemented with 0.5% glucose (GM17). Cells were grown until the late-exponential phase of growth (OD600 ~1.5 – 2) and harvested for chromosomal DNA isolation, as described before [28]. DNA concentrations were determined spectrophotometrically [29].

### Comparative genome hybridization

Chromosomal DNAs (1 – 2 μg) of *L. lactis *IL1403 and *L. lactis *MG1363 were randomly labeled and linearly amplified using the BioPrime DNA Labeling System (Invitrogen, Breda, the Netherlands) according to the manufacturers' instructions, with the following modifications. For labeling, 5 μL 10× dNTP-mix (1.2 mM dATP, dGTP and dCTP; 0.6 mM dTTP; in 10 mM Tris, 1 mM EDTA, pH 8.0), 3 μL Cy3- or Cy5-dUTP (Amersham Bioscience Europe, Freiburg, Germany) and 1 μL Klenow enzyme were used. The DNA-primer hybrid elongation and labeling reaction was performed by incubation for 2 h at 37°C in a Bio-Rad iCycler Thermal Cycler (Bio-Rad Life Science, Veenendaal, the Netherlands). The Cy-dye-labeled DNA fragments were purified using the QiaGen QiaQuick Nucleotide Removal Kit (Qiagen Benelux BV, Venlo, the Netherlands). Finally, the labeled purified DNA fragments of *L. lactis *MG1363 and *L. lactis *IL1403 were mixed and hybridized to *L. lactis *IL1403 DNA microarrays containing features representing 2126 open reading frames (ORFs) in duplicate. DNA-microarrays were prepared from amplicons of 2126 genes selected from the 2266 annotated genes (ORFs smaller than 80 bp were omitted) in the genome of *L. lactis *ssp. *lactis *IL1403 [25]. The oligo-nucleotides used to generate these amplicons were designed using the in-house developed programs Unifrag and GenomePrimer [30]. To reduce cross-hybridization between array feature and target DNA sequences the amplicons have sizes of 80 – 800 bp (depending on the gene sizes; with 403 amplicons being smaller than 300 bp) and comprise the most unique part of a gene. Paralogous genes of which no unique region could be identified were omitted. The amplicons were synthesized by EuroGentec (Seraing, Belgium). Further details have been described before [31]. After overnight hybridization at 40°C, slides were washed in [2× SSC, 0.5% SDS] at room temperature and subsequently for 5 min in [1× SSC, 0.25% SDS] to remove non-specifically hybridized DNAs. Slide scanning and spot intensity quantization were done as described before [31].

### Generation of likely conserved gene sets

In this study, we take advantage of the fact that both genome sequences of the strains hybridized to the microarray are known. To assess the performance of S-Lowess compared to other methods, we performed alignments of the 2126 amplicon sequences of *L. lactis *IL1403 to the ORFs of *L. lactis *MG1363 [32] using BLAT [23]. Next, the alignments were categorized in 12 different LCG sets based on sequence identity (90%, 95% and 99%) and alignment length (50, 100, 150 or 200 bp). Five additional LCG sets were generated on the basis of BLAT E-value cutoffs (1, 1E-100, 1E-200, 1E-300 and 1E-400).

The performance of S-Lowess was determined in a realistic case, where only one genomic sequence of the two strains hybridized to the microarray is known (in this study that of *L. lactis *IL1403). In this case we assume that the genome sequence of *L. lactis *MG1363 is unknown. Still we would like to use S-Lowess on the microarray data. We first select a number of reporter genomes, which are considered to be close to the unknown genome (in this case that of *L. lactis *MG1363). In this study, three *Streptococcus *strains were selected as reporter genomes. We want to know which *L. lactis *IL1403 amplicons are likely to be conserved in the genome of MG1363. By comparing the IL1403 amplicons to the ORFs in the three *Streptococcus *strains, we can select a subset of amplicons (LCG set) that are conserved in the *Streptococcus *strains. Since the assumption is that *L. lactis *MG1363 and the *Streptococcus *genomes are closely related, the amplicons of this LCG set should also be conserved in *L. lactis *MG1363. The LCG set consisting of 57 genes was generated by aligning *L. lactis *IL1403 amplicon sequences to the ORFs of three related organisms: *Streptococcus pneumoniae *TIGR4, *S. pneumoniae *D39 and *S. pneumoniae *R6. An amplicon was considered to be likely conserved to the *L. lactis *MG1363 counterpart when the following two criteria were met: it has (i) a hit in each of the three genome sequences and only one hit in each genome sequence itself, and (ii) at least 80% nucleotide identity over 100 bp.

### Prediction of deletions in *L. lactis *MG1363

To benchmark the performance of S-Lowess with different LCG sets and the normalization methods described here, the existing knowledge of the presence of genes in both genome sequences of *L. lactis *IL1403 and *L. lactis *MG1363 was used.

The difference in gene content of the *L. lactis *MG1363 and *L. lactis *IL1403 genomes was determined during the genome annotation phase of *L. lactis *MG1363 and is based by analyzing ORF similarity with BLASTN, gene context, and by manual curation [32]. Of the MG1363 ORFs that can be detected by the amplicons present on the *L. lactis *IL1403 DNA microarrays, only a few are duplicated compared to IL1403. Since both genomes have limited sequence identity (on average 85%), many *L. lactis *MG1363 ORFs have a low sequence identity with *L. lactis *IL1403 counterparts and will show decreased hybridization efficiencies in the aCGH experiment described here. Since the aCGH methodology cannot distinguish between absent genes and genes with lower (< 80 –   90%) sequence identity we therefore only show the results for genes that are annotated as missing in MG1363 compared to IL1403. Chromosomal phage genes present in both organisms were removed prior to the comparison because a large number of duplicated phage genes have relatively small differences in nucleotide sequence and would therefore show strong cross hybridization.

### aCGH data normalization and statistical analyses

In this study, 4 aCGH comparisons (slides) between *L. lactis *MG1363 and *L. lactis *IL1403 were performed (including dye swap with biological replicates; see also [17]). The resulting aCGH slide signals were normalized using the different LCG sets (see above) yielding ratios of signals of labeled gDNA of MG1363 over those of IL1403. A maximum of 8 ratios per amplicon (gene) were obtained for the 4 hybridized slides (each with 2 replicate spots per amplicon). Only array features with at least 5 measurements were used in this study. The normalization methods evaluated in this study are: a) no normalization, b) total signal normalization, c) grid-based Lowess (implemented in PreP; f = 0.7) [24], and d) S-Lowess using different subsets of conserved lactococcal genes (for details see above) with f = 0.7. Results with the MANOR R package (spatial normalization; standard parameters) [12,15] are only shown in Figure S2 [17] since the normalized ratios were reproducible for very few genes only and complete analyses could not be performed. The GENCOM method [18] uses a convergence algorithm to determine conserved genes from the aCGH data. It uses an internal statistical measure for determining divergent genes; therefore the normalized expression values are not available. The GENCOM results can be viewed at [17].

For Figure 3, genes were considered to be absent using mild cutoffs: a normal ratio (IL1403 over MG1363 signal) > 1.5 and a Cyber-T [33] Bayesian p value of < 0.01.

All aCGH data used in this study can be viewed at [17].

### Availability

The S-Lowess tool is accessible from [34]. The aCGH dataset of *L. lactis *IL1403 and *L. lactis *MG1363 (accession number GSE3039) is available at the GEO repository .

## Authors' contributions

SvH conceived the study and drafted the manuscript. SvH and AZ generated the figures. HK and RB conducted the aCGH experiments. SvH, AZ and RB performed the *in silico *genome comparisons. VR and OT designed and programmed the Windows PreP and command-line PreP software. SvH designed and programmed the web-interface and LCG determination. JK and OPK supervised the microarray work and the subsequent data analyses. All authors read, corrected and approved the final manuscript.
